# Once upon a time, inflammation

**DOI:** 10.1590/1678-9199-JVATITD-2020-0147

**Published:** 2021-04-09

**Authors:** Jean-Marc Cavaillon

**Affiliations:** 1 French National Research Agency(ANR), Paris, France.

**Keywords:** Historical review, Inflammation, Fever, Phagocytosis, Diapedesis, Antiseptics, Antibiotics

## Abstract

Inflammation has accompanied humans since their first ancestors appeared on Earth. Aulus Cornelius Celsus (25 BC-50 AD), a Roman encyclopedist, offered a still valid statement about inflammation: “*Notae vero inflammationis sunt quatuor: rubor et tumor cum calore and dolore*”, defining the four cardinal signs of inflammation as redness and swelling with heat and pain. While inflammation has long been considered as a morbid phenomenon, John Hunter (18^th^ century) and Elie Metchnikoff (19^th^ century) understood that it was a natural and beneficial event that aims to address a sterile or an infectious insult. Many other famous scientists and some forgotten ones have identified the different cellular and molecular players, and deciphered the different mechanisms of inflammation. This review pays tribute to some of the giants who made major contributions, from Hippocrates to the late 19^th^ and first half of the 20^th^ century. We particularly address the discoveries related to phagocytes, diapedesis, chemotactism, and fever. We also mention the findings of the various inflammatory mediators and the different approaches designed to treat inflammatory disorders.

## Introduction

Inflammation is older than humanity itself and the earliest signs of inflammatory processes can be found on dinosaur bones. Consequently, inflammation has always accompanied humans since they are on Earth as it can be seen on the bones of the first humanoids and of *Homo sapiens*. The first precise diagnoses of inflammatory disorders were made on Egyptian mummies by Sir Marc Armand Ruffer (1859-1917). Accompanying Sir Grafton Elliot Smith (1871-1937) and Frederic Jones Wood (1879-1954), the first British Egyptologists and anthropologists, he gave birth to a new science: “paleopathology”. He carried out pioneer post-mortem diagnoses of arthritis and spondylitis, and by studying the mummy of Ramses II, he diagnosed that the pharaoh had suffered from atherosclerosis [1].

## Defining inflammation - the early reports

**Figure 1.**
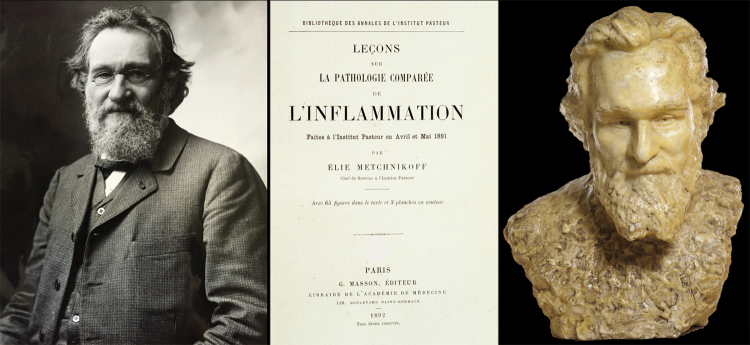
Elie Metchnikoff (1845-1916) conducted major investigations on inflammation. He and his collaborators defined phagocytosis, discovered alveolar macrophages, microglial cells, observed opsonisation, chemotactism, efferocytocis pinocytosis and netosis. Left: photo of Elie Metchnikoff taken by Nadar in 1905. Center: His lectures on inflammation at Institut Pasteur. Right: Sculpture made by Olga his wife. Copyright by Institut Pasteur/Musée Pasteur.

One of the earlier descriptions of inflammatory processes is provided in Edwin Smith papyruses. These Egyptian papyruses (around 1520 BC), copies of even older ones (3400 BC), depict 48 cases of injury, trauma and even surgery. One of the very firsts to define the parameters of inflammation was Aulus Cornelius Celsus (25 BC-50 AD), a Roman encyclopedist to whom we owe the famous statement: “*Notae vero inflammationis sunt quatuor: rubor et tumor cum calore and dolore*” (The signs of inflammation are four: redness, swelling, fever and pain). A fifth element was later added “loss of organ function”. Erroneously attributed to Galen of Pergamum (129-201 AD) [2], it could have been proposed by either Thomas Sydenham (1624-1689) or Rudolf Virchow (1821-1902). Of course, inflammation has been for a long time considered a morbid response of the host to any types of insults. However, John Hunter (1728-1793), a Scottish surgeon, appropriately defined inflammation in his book published one year after his death: “*Inflammation in itself is not to be considered as a disease, but as a salutary operation, consequent either to some violence or some disease*” [3]. Despite this appropriate definition, one could still read in 1865 in the French dictionary of medicine that “*Inflammation is a complex morbid phenomenon, particularly associated with the function of blood circulation*”. In his lectures on inflammation, Elie Metchnikoff (1845-1916) stated in 1891 that phagocytes were the participant of the inflammatory process and that inflammation should no longer be seen as only being deleterious [4] (Figure 1). Since Metchnikoff, many mechanisms accompanying inflammation have been further deciphered and mediators have been characterized.

## Defining the role of phagocytic cells

### Phagocytosis

In 1882, Elie Metchnikoff (1845-1916), when he was in Messina (Sicilia) with his family-in-law, made his key observation. He stuck rose thorns into starfish larvae and was surprised to see that many phagocytic cells in the hemolymph of the starfish surrounded the “foreign object”. He also observed the process in Daphnia, which he infected with yeast [5]. These cells were able to move, ingest, and destroy the yeast cells within the water fleas. He then understood that this process was an important mechanism of host defense against infectious agents. When he met his Austrian friend Karl Claus (1835-1899) in Vienna, he was offered to publish his observation in a Viennese journal and Karl Claus coined the word “phagocyte” (1883) [6]. Metchnikoff was awarded with the Nobel prize in 1908 for his discovery of phagocytosis, and he is considered as the father of the cellular innate immunity (in addition to be the father of probiotic, microbiota and gerontology) [7,8].

Interestingly, he was not the first one to observe the phenomenon. The fact that animalcules like amoeba could engulf some nutrients or even other alive individuals such as bacteria had been observed under the microscopes by numerous scientists: in Denmark by Otto Friedrich Müller (1730-1784) in 1773; in Germany by Johann August Ephraim Goeze (1731-1793) in 1777, and Wilhelm Friedrich von Gleichen-Russwurm (1717-1783) in 1778; in England by Andrew Pritchard (1804-1882) in 1834; in Switzerland by Rudolph Albert von Kölliker (1817-1905) in 1849, and Édouard Claparède (1873-1940) in 1854; and in USA by Joseph Mellick Leidy (1823-1891). In 1847, Alexander Ecker (1816-1887) in Germany had described erythrocytes inside rabbit spleen cells [9], an observation confirmed in 1870 by Nathanael Lieberkühn (1821-1887) who reported that leukocytes could ingest erythrocytes [10]. In 1871-1873, Giulio Bizzozero (1846-1901) provided the first drawings of macrophages that had ingested erythrocytes [11]. He stated that reticular cells could ingest infective particles that were carried by the lymphatic liquid. He made this farsighted statement: “*( this fact is, perhaps, the cause of the stoppage of some infections to the lymphatic glands which are connected to the part covered by the infection through the lymphatic vessels*” [12]. In 1875, Sir William Osler (1849-1919) reported that alveolar macrophages of coal miners were full of carbon particles [13]. In 1881, Alexander Ogston (1844-1929) published cartoons of groups of cocci, mostly free, but sometimes in or on large nonnucleated masses of protoplasm of leukocytes [14]. 

While Metchnikoff was in Messina trying to understand the aim of phagocytosis, Robert Koch (1843-1910) identified *anthrax bacilli* within white blood cells. Yet, Koch had interpreted his finding to mean invasion of the host by bacterial pathogens [15]. However, the discovery of phagocytosis by Metchnikoff was not easily accepted. He recalls: *“( The controversy about the phagocytosis could have killed me, or sooner permanently weakened me. Sometimes, (I remember such attacks of Lubarsch in 1889, and those of Pfeiffer in 1894) I was ready to get rid of life*” (Oct. 1913). But some other scientists were fully convinced. For instance, Sir Marc Armand Ruffer was an ardent advocate of phagocytosis in the UK. In 1892, he wrote this nice metaphor in the *British Journal of Medicine*: “*Should anyone meet a dead lion and find a lamb inside, he, knowing the habits of the lion would not conclude that the lamb had taken refuge in that. True, after a surfeit of lamb, the lion might die of indigestion but the chance of the lamb ever getting out alive would be very small*” [16].

Other sources of phagocytes were also discovered. In Metchnikoff’s laboratory, Nicolas Tchistovitch (1860-1926) described in the lungs that what were erroneously considered as epithelial cells were in fact alveolar macrophages [17]. And Metchnikoff, himself described what he called “neuronophages”, which were phagocytic cells within the central nervous system, now known as microglial cells [18]. Carl Wilhelm Kupffer (1829-1902) described stellate cells in the liver. He incorrectly believed that these cells were an integral part of the endothelium of the liver’s blood vessels. In 1898, Tadeusz Browicz (1847-1928), a Polish pathologist, correctly identified them as macrophages, now known as Kupffer cells [19,20]. In 1922, Karl Albert Ludwig Aschoff (1866-1942) introduced the term “reticulo-endothelial system” that included endothelial cells, fibrocytes, histiocytes, and splenocytes, monocytes and Kupffer cells [21]. However, this concept was of limited help since it mixed cells that undergo pinocytosis and cells that are able to achieve phagocytosis. Of note, the word “pinocytosis” was coined in 1894 by George Gabritchevsky (1860-1907), a Muscovite researcher who spent some time in Metchnikoff’s laboratory [22]. Pinocytosis, is a mode of endocytosis in which molecules present in extracellular fluid are internalized into the cell through an invagination of the cell membrane, resulting in a suspension of the molecules within a small vesicle inside the cell.

### Opsonisation

In 1895, the phenomenon of opsonization was discovered by two Belgian scientists. Jules Bordet (1870-1961) who was working in Metchnikoff’s laboratory reported the capacity of immune sera to induce the agglutination of bacteria and their lysis. While he used the term “alexin”, a word coined by Hans Ernst August Buchner (1850 -1902) to define the action of the complement system, he called "stimuline", the action the sera has to favor the phagocytosis of bacteria by macrophages [23]. Joseph Denys (1857-1932), a professor of bacteriology and anatomy at Louvain University made a similar observation. He stated: “*In vaccinated rabbit, leukocytes get from sera their power to engulf and destroy Streptococcus pyogenes*” [24]. In 1903, Sir Edward Almroth Wright (1861-1947), a British bacteriologist and immunologist who discovered an effective vaccine for typhoid fever, coined the word “opsonisation” from Greek “οπσονο” meaning “I prepare victuals for(”. He nicely explained: “*The body fluids modify bacteria in a manner which renders them a ready prey to phagocytes*” [25]. In 1904, Friedrich Neufeld (1869-1945), a physician and bacteriologist, director of the Robert Koch Institute in Berlin, described the exactly same phenomenon, only based on much more solid experimental works [26]. However, the word “bacteriotropin” that he employed did not survive the effects of time, and was not used after 1930.

### Efferocytosis

In 2003, Aimee M. deCathelineau and Peter M. Henson coined the word “efferocytosis” derived from the Latin prefix *effero*, meaning “to take away, to put away, to carry to the grave, or to bury” [27]. However, Metchnikoff already knew the process as he had observed it: “[(] *many of phagocytes perish and are taken in by other phagocytes, as can be seen in every case a few days after the onset of the inflammation*” [28]. However, it was described for the first time by Giulio Bizzozero in 1871-1872, studying eye inflammation: “*In summary, my observation showed the presence of big cells able to engulf white blood supurative cells or red blood cells in their contractile protoplams. This represents a way through the pus or blood is absorbed from the anterior chamber*” [29]*.* Marc Armand Ruffer, while working with Metchnikoff, also described the phenomenon in 1890: “*Macrophages are able to swallow microphages (neutrophils) and to destroy and digest them*” [30]*.*

### Netosis

Octave Gengou (1875-1957), Jules Bordet’s brother-in-law, in Metchnikoff’s laboratory, was chasing the origin of the bactericidal property of sera and concluded that it could derived from leukocytes [31]. His colleague, in the same laboratory, Constantin Levaditi (1874 - 1953) from Bucharest came to the conclusion that: "*Altered in their vitality, deteriorated, destroyed, neutrophils still contribute to anti-bacterial immunity*” [32]. In 2004, Arturo Zychlinky and his colleagues described the process of netosis, and demonstrated that the release of intracellular content by dying neutrophils can contribute to kill the pathogens caught in these NETs (neutrophil extracellular traps) [33].

### Diapedesis and chemotactism

Galen of Pergamon (129-201) had considered that the formation of pus was part of the healing process (“*pus bonum et laudabile*”, good and commendable pus). Pus was supposed to facilitate the removal of the unhealthy mood of the injured body. Galen’s theory prevailed for more than a millennium until Ugo Borgognoni de Lucca (1160-1257), a surgeon and the founder of the Faculty of Medicine of Bologna, and his son Theodorico Borgognoni de Lucca (1205-1298), clergyman and surgeon, critiqued the doctrine in a four-volume work entitled “Chirurgia” (1267). In 1810, Alexandre François Ollivier (1790-1844), a physician accompanying Napoleon’s campaign, injected himself with the pus from a wound of a severely injured soldier dying from putrid fever and was the first one to demonstrate its contagiousness [34]. He had to use cauterization to stop the progression of the infection.

In 1845, two scientists studied pus under the microscope and observed that the cells present in the pus were similar to the cells found in blood. Alfred Donné (1801-1878), a French bacteriologist and physician, studied blood from a leukemic patient and thought that it might contain pus cells, although he observed a clear-cut difference between these cells [35]. In contrast, William Addison (1802-1881), the physician to the Duchess of Kent, appropriately understood that pus cells were derived from white blood cells (or colorless as they called them): “*Colorless blood cells are deposited all over the interior of the vessels;* [(] *and at length pass into the tissue;* [(] *These facts are all independent of any orifices, rupture or pores in the vessels, allowing the escape of cells*” [36]. Yet, the observation of diapedesis was not made.

Augustus Volney Waller (1816-1870), a British neurophysiologist, was the first to observe natural leukocyte emigration in 1846, using the frog tongue. However, the origin of pus cells was not fully demonstrated [37]. In 1858, Rudolf Virchow (1821-1902), a medical doctor at Charité Hospital in Berlin, the father of the cell theory, erroneously believed that pus cells were derived from tissue elements following cell division [38]. Nevertheless, his assistant and two of his students made the final demonstration. In 1863, Friedrich Daniel von Recklinghausen (1833-1910), a physician and pathologist who left Virchow for a position of professor at the University of Würzburg before joining the University of Strasbourg, induced an experimental keratitis in frogs with silver nitrate. He characterized the pus cells in humor aqueous during acute inflammation and made the seminal observation of the contractility and mobility of colorless cells [39]. 

In 1867, Julius Friedrich Cohnheim (1839-1884) used a combination of colloidal aniline blue injections and microscopy to prove, what Addison had hypothesized, that white blood cells cross blood vessels to become pus cells [40]. And in 1875*,* Julius Arnold (1835-1915), using an injection of cinnabar to demonstrate the borders of endothelial cells, came to the conclusion that leukocytes move across blood vessel walls (diapedesis) by passing between endothelial cells at either points of dense staining, “stigmata”, or circles of stain, “stomata” [41]. Quite surprisingly, 33 years after Addison’s work, Louis Pasteur (1822-1895) wrote in 1778: “*For us currently, it would be the red blood cells that would be the pus cells from a simple transformation from the first into the second*” [42]. 

This illustrates that great scientists can also make wrong statements and be poorly aware of previous discoveries! Nowadays, it is well understood that diapedesis of circulating cells toward tissues is under the control of chemoattractant signals. Interestingly, the concept came from an observation made with bacteria. In 1884, Wilhelm Pfeffer (1845-1920), a botanist at the University of Tübingen, Germany, observed bacteria swimming toward the vicinity of the tip of a capillary tube filled with nutrient sugar that had been dipped into a bacterial culture broth. He coined the word “chemotaxis” [43]. The *in vivo* phenomenon was first reported in 1889 by Cornelis Adrianus Pekelharing (1848-1922), a Dutch physician and professor of physiological chemistry and histology at the University of Utrecht [44]. He put cotton wool soaked with anthrax bacilli in the peritoneal cavity of a frog. Retrieving this cotton wool some time later, he showed that it contained significantly more leukocytes than those that had been soaked with neutral liquid. He came to the conclusion that bacteria produce chemoattractant factors. 

In 1890, Gabritchevsky drew the same conclusion [45]. He inserted under the skin of frogs, rabbits or axolotl, small capillary tubes filled with alive or dead bacteria. Twenty-four hours later, the capillary tubes were full of leukocytes, whereas this was not the case if the capillaries had been filled with saline. The same year in the laboratory of Paul Héger (1846-1925) at the Solvay Research Institute (Brussels, Belgium), Jean Massart (1865-1925), a doctor in sciences and medicine working with Charles Bordet, the brother of the famous Jules Bordet who got the Nobel Prize for his discovery of the complement, was the first to demonstrate that the host can make chemoattractant factors [46]. He injected bovine bile subcutaneously in a frog. Then, he sampled the transudation liquid and transferred it in a capillary tube into the abdominal cavity of another frog. Twenty hours later, the capillary tube was full of leukocytes, whereas this was not observed with a capillary tube filled with normal lymph. Thus, he had demonstrated that chemoattractant factors were produced by the frog. 

In 1891, the same team performed another elegant demonstration [47]. They injected s.c. bacteria (*Micrococcus prodigiosus*, nowadays known as *Serratia marcescens*) in a rabbit. Thirty-five minutes later, they sampled the blood, prepared the serum, placed it in a capillary tube and finally transferred it into the peritoneal cavity of another rabbit. Eight hours later, the capillary tube was full of leukocytes, whereas this was not the case when the tubes were filled with normal serum. For the first time, they had demonstrated that chemoattractant factors were produced in response to infection. In 1938, Valy Menkin (1901-1960) purified a substance from inflammatory exudates that induced an increased capillary permeability followed by a migration of polymorphonuclear leukocytes. He called his factor “leukotaxine” [48]. Unfortunately, despite all his efforts, he could not succeed, and further studies revealed no convincing evidence that “leukotaxine” exist as a distinct chemical entity [49]! The discovery of well-identified chemoattractant factors, later called chemokines (the word was coined in 1992), was made in 1987 and 1988 with the description of interleukin-8 [50] and macrophage inflammatory protein [51], renamed CXCL8 and CCL3, respectively, according to the revised nomenclature of chemokines [52].

## Fever

For a while it was believed that fever was consecutive to some obstructions within the blood vessels leading to an accelerated movement within the free vessels. At the beginning of the 18^th^ century, a defender of this concept was the Italian physician Lorenzo Bellini (1643-1704) [53]. Herman Boerhaave (1668-1738), a Dutch physician, also thought that increased heartbeats were the source of the accelerated circulation and fever [54]. In 1744, François Boissier de Sauvages de Lacroix (1706-1767), while translating in French the book on “haemastatic” written by Stephen Hales (1677-1761), added his personal view on fever, confirming the prevailing concept that inflammation was associated with increased blood flow [55]. Thanks to scientists and doctors such as John Davy (1790-1868) in the United Kingdom who made the first sets of temperature measurements in different humans and in different environments (1816-1818) [56], Antoine Becquerel (1852-1908) in France who invented the pyrometer to measure human temperature (1835) [57], Thomas Clifford Allbutt (1836-1925) who invented the clinical thermometer (1866) [58], and Karl August Wunderlich (1815-1877) of Germany who made more than one million measurements on more than 25 000 patients (1868), normal temperature and fever could be definitively and precisely defined [59].

William H. Welch (1850-1934), the first dean of the Johns Hopkins School of Medicine, offered a wonderful definition of fever in his 1888 Cartwright lecture: “*The real enemy in most fevers is the noxious substance which invades the body, and there is nothing to prevent us from believing that fever is a weapon employed by Nature to combat assaults of this enemy. According to this view, the fever-producing agents light the fire, which consumes them. It is not incompatible with this conception of fever to suppose that the fire may prove injurious also to the patients and may require the controlling hand of the physician*” [60]. What could these “noxious substances” be? What was the relationship between putrescent material, bacteria and toxins and their respective capacities to induce fever [61]? Nikolai Fedorovich Gamaleïa (1859-1949), who worked in Odessa and Moscow, spent some time with Metchnikoff at the Institut Pasteur and reported that an injection of dead bacteria could induce fever in rabbits and sheep. Most fascinatingly, he showed that filtered alcoholic extracts of spleen from pyretic sheep could induce fever within 30 min in rabbits [62]. 

Although Gamaleïa was foreseen to join the young institute, it finally never occurred [63]^1^. The following year (1889), Marc Armand Ruffer, who had also joined Elie Metchnikoff and Louis Pasteur shortly after the Institute was set up, published a paper with Albert Charrin (1856-1907) who was a professor at the prestigious College de France. They reported that the filtered culture of pyocyanic bacillus (*Pseudomonas aeruginosa*) could induce fever in rabbits, in the absence of alive or dead bacteria [64]. Of note, this report was published three years before the German physician and bacteriologist Richard Pfeiffer (1858-1945) coined in 1892 the word “endotoxin” that is nowadays widely used. Most fascinating, the authors wrote, although did not prove, that fever was the consequence of the activation of macrophages. In 1894, Eugenio Centanni (1863-1942), an Italian pathologist, recognized the intimate relationship between the pyrogenic and toxic properties of the bacterial poison, which he found to be chemically inseparable. This led him to name his material “pyrotoxina” [65]. In 1890, Hans Ernst August Buchner (1850-1902) had shown that this material was also pyogenic [66]. Valy Menkin (1901-1960) was the first to attempt to purify the endogenous mediator that could be responsible of fever. In 1943, he isolated a mediator called “pyrexin” [67]. Unfortunately, further analysis of his work suggested that his factor was contaminated with endotoxin [68].

Subsequently, in 1953, Ivan L. Bennet Jr. (1922-1990) and Paul Beeson (1908-2006) were the first ones to extract a fever-producing substance from rabbit polymorphonuclear leukocytes [69]. In 1955, Elisha Atkins (1921-2005) and W. Barry Wood Jr. (1910-1971) isolated a circulating endogenous pyrogen in the blood after the injection of typhoid vaccine [70]. In 1984, Charles Dinarello’s team cloned the human interleukin- β(IL-1β), known to be the endogenous pyrogen among many other activities [71]. Other pyrogenic cytokines have been described such as the tumor necrosis factor (TNF) and the IL-6. Acting within the central nervous system, both IL-1β and TNF induce IL-6, which on his turn induces the release of prostaglandin E2 (PGE2) [72-74]. Of note, some chemokines, such as IL-8 (CXCL8), MIP-1 (CCL3) and RANTES (CCL5) also behave as endogenous pyrogen [75-77].

## The discoveries of inflammatory mediators

If nowadays the complement system is known to contribute to inflammation, particularly through the release of anaphylatoxins, its first identification was associated with its capacity to induce bacteriolysis and hemolysis in partnership with antibodies. In 1886, Joseph von Fodor was the first to report the bactericidal activity of the blood [78] and in 1891 Hans Buchner (1850-1902) coined the word “alexin” [79] while the word “complement” was proposed in 1899 by Paul Ehrlich (1854-1915) [80] who shared the 1908 Nobel Prize with Metchnikoff. Once Jules Bordet (1870-1961) had joined the laboratory of Elie Metchnikoff in 1894, he started to investigate the bactericidal properties of the sera, and reported similarities with the process of hemolysis by anti-red blood cells antisera [81]. Bordet was awarded the 1919 Nobel Prize for his investigation on the complement system and its coordinated action with the antibodies.

Surprisingly, when the word anaphylatoxin was employed in a paper reporting a model of anaphylatoxic shock, its existence was denied. Indeed, an unanticipated severe reaction had been observed in 1901 when Charles Richet (1850-1935, Nobel Prize 1901) and Paul Portier (1866-1962) involuntarily killed dogs after the second injection of a hypnotoxin from physalia**.** One of the main mediators of anaphylatoxic shock is histamine. Histamine, released by activated mast cells favors vasodilatation, fall in blood pressure, increased vascular permeability and mucus secretion. The molecule was synthesized for the first time in 1907 by Adolf O.R. Windaus (1876-1959, Nobel Prize 1928), and Henry H. Dale (1875-1968) (Nobel Prize 1936) who discovered its activity on smooth muscle and on endothelium. In 1937, Anne-Marie Staub (1914-2012), who worked in the laboratory of Daniel Bovet (1907-1992, Nobel Prize 1936) at Institut Pasteur, was the first scientist to save an animal from a shock induced by histamine using thymoxyethyldiethylamine (compound 929F) and phenolic ethers as antihistaminic drug [82]. However, the employed products were toxic, and the first antihistaminic to be used in humans (Antergan) was developed by Bernard Halpern (1904-1978) at the Rhône Poulenc pharmaceutical company [83].

In 1930, William S. Tillett (1892-1974) and Thomas Francis Jr. (1900-1969), working at the Rockefeller Institute in New York, identified in the serum of a rabbit injected with *Streptococcus pneumoniae* an interaction activity with a carbohydrate extract of the bacteria (called “fraction C”). The “C-reactive protein” (CRP) was thus discovered and shown to be present in the inflammatory settings independent of any *S. pneumoniae* infection [84]. CRP was purified by Colin M. MacLeod (1909-1972) and Oswald T. Avery (1877-1955) in 1941 [85] and crystallized in 1947 by Maclyn McCarty (1909-1972) [86]. All these investigators were working at the Rockefeller Institute. The pentameric structure was determined in 1977 and its liver origin was demonstrated the following year. CRP is an acute phase protein released by the liver in response to inflammatory cytokines, particularly IL-6. CRP contributes to the elimination of damaged cells and display anti-inflammatory properties on neutrophils while in contrast it has pro-inflammatory action on the endothelium. Nowadays, CRP is one of the most common biomarker to confirm the presence of an inflammatory reaction.

In 1935, Ulf von Euler (1905-1983) discovered the prostaglandins which contribute to vasodilatation (erythema), vascular permeability, pain, edema (swelling) and fever. This Swedish physiologist and pharmacologist, who also discovered noradrenalin and substance P, was awarded the Nobel Prize in 1970. His father, Hans von Euler-Chelpin (1873-1964) was also awarded the Nobel Prize in 1929 for his contribution to the fermentation of sugar and his mother, Astrid Cleve (1875-1968), a botanist, geologist, and chemist at Uppsala University, had been the first woman in Sweden to obtain a doctoral degree of science. For their studies on prostaglandins, related biologically active substances, and the discovery that aspirin prevented the production of prostaglandins [87], the Swedish Sune K. Bergström (1916-2004) and Bengt I. Samuelsson (1934-) and the British Sir John R. Vane (1927-2004) were awarded the 1982 Nobel Prize, respectively.

Glucocorticoids were discovered in 1936-1941 by three scientists who were awarded the Nobel Prize in 1950 for their discoveries related to the hormones of the adrenal cortex, their structure, and biological effects. These studies ended to the discovery of the glucocorticoids that are potent mediators to turn off the inflammatory process. In the United States, Philip Showalter Hench (1896-1965) postulated that a natural mediator, which he called substance X, was involved in the improvement of arthritis when patients developed jaundice. He collaborated with Edward Calvin Kendall (1886-1972), who purified a large number of substances from adrenals. Among those, was the compound E (dehydrocorticosterone) that was finally considered as substance X [88]. In Switzerland, Tadeusz Reichstein (1897-1996), who was born in Poland, also produced numerous substances from adrenals, of which few were bioactive. In 1937, he reported the production of desoxycorticosterone and its first clinical essays [89].

After the identification of the endogenous pyrogen in 1953 as aforementioned, the next cytokine being described was the interferon. Interferons possess antiviral activity but is also known to modulate the inflammatory reaction. In 1954, two Japanese scientists Yasu-ichi Nagano (1906-1998) and Yasuhiko Kojima (1928-) noticed that rabbit skin or testis previously inoculated with UV-inactivated virus exhibited inhibited viral growth when reinfected at the same site with live virus. They hypothesized that this was due to some inhibitory factors. They made two major mistakes. The first one was to publish in a French-speaking journal and the second one was not to create a neologism [90]. This explains why the British Alick Isaacs (1921-1967) and the Swiss Jean Lindenmann (1924-2015), who published three years later in English and coined the word “interferon”, have been regularly considered as the discoverers of this cytokine.

In 1966, a new cytokine was discovered by Bloom and Bennett who illustrated the capacity of the supernatants of T cells involved in delayed type hypersensitivity to prevent macrophage migration (“macrophage migration inhibitory factor”, MIF) [91]. Most fascinatingly, this cytokine was rediscovered later as a product of the pituitary gland. Interestingly, MIF brought Stanley Cohen to create in 1974 his neologism “cytokine” [92]. Then, the words lymphokine and monokine were commonly used, but Cohen had shown that fibroblasts infected with viruses could release MIF. Thus, it was obvious that these mediators were not only the products of immune cells but also an universal language of the cells (see my interview of S. Cohen to commemorate the 30^th^ anniversary of the birth of the word "cytokine"[93]). Endogenous pyrogen, osteoclast activating factor, hemopoietin-1, catabolin, lymphocyte-activating factor, epidermal cell-derived thymocyte-activating factor are various mediators identified between 1953 and 1981, which appeared to be the diverse biological properties of one molecule, namely, interleukin-1. It was later categorized into IL-1α and IL-1β, which are members of a family of 11 molecules, including agonists with different properties, and antagonists [94, 95]. IL-1 bioactivity was the first to be identified in an inflammatory fluid. In 1982, a French dentist working in Joost Oppenheim’s laboratory demonstrated the presence of IL-1 in the gingival fluid of patients with periodontal inflammation [96]. In 1986 in Norway, Waage *et al.* [97] reported for the first time the presence of TNF in the plasma of patients with severe meningococcal sepsis. Later on, all pro- and anti-inflammatory cytokines could be detected in the plasma of patients with severe sepsis or sterile systemic inflammation [98]. These measurements illustrate the concept of cytokine storm observed in many severe inflammatory settings, but those are just the tip of the iceberg [99]. The term cytokine storm has been employed to characterize the excess of cytokine production, which can be monitored in the blood compartment. However, as illustrated with the COVID-19 setting, the cytokine storm can also be found within tissues, such as the lungs. Most fascinating is this statement attributed to William Osler in 1904: “*Except on few occasions, the patient appears to die from the body’s response to infection rather than from it*”. It fully recognizes the yin yang properties of a large number of molecules, particularly the cytokines, which may be both protective or deleterious, while their classification as pro- or anti-inflammatory appears far too simplistic [100].

## Treating inflammation

In fact, long before inflammatory processes could be understood or even defined, various therapeutic approaches had been proposed to treat different types of inflammatory diseases.

### Herbology

According to Chinese mythology, herbology or the use of plants to cure diseases was introduced by Emperor Shennong in 2800 BC, and the first ever book on medicinal plants was published in China in 300-200 BC. Other testimonies of interest on plants to cure diseases or at least to relieve pain and fever were provided by Edwin Smith and Georg Moritz Ebers papyruses. Not only did they describe case reports of injuries but also listed different plants to be used against various types of injuries (crocodile bites, burns, fractures, bowel diseases, joint pains, etc). For example, infusion of dried myrtle was recommended for rheumatic pain. The interest to use plants to cure inflammatory diseases was perpetuated by the Greeks, and Hippocrates (450-370 BC) used extracts from willow bark to relieve pain and fever. The study of willow bark ended with the discovery of aspirin in the 19^th^ century. In addition, Hippocrates and Theophrastus (circa 371-288 BC) used the opium poppy as treatment against pain. Pedanios Dioscoride (circa 40-circa 90), a Greek physician, is famous for his herbarium known under the name of *"De Materia Medica"*, a description of more than six hundred plants and almost one thousand remedies. It remains the main source of knowledge of medicinal plants during Antiquity. In 1535, Leonhart Fuchs (1501-1566), a renowned Bavarian physician, was called to Tübingen by the Duke of Württemberg to participate in the reform of the university. There, he established a medicinal plant garden, one of the oldest in the world. He wrote *"De historia stirpium commentarii insignes*” (Distinguished commentaries on the history of plants) published in Basel in 1542. Fuchs describes German flora and foreign species. He was mainly inspired by Dioscorides propagating the ancient and medieval tradition. Finally, it is worth mentioning Bernardino Ramazzini (1633-1714) and Francesco Torti (1658-1741), physicians of the Duke of Modena who introduced the use of quinine-rich cinchona bark to treat malaria. Ramazzini recognized its importance: “*Quinine did for medicine what gunpowder did for war* ”.

### Aspirin

In 1828, Johann Buchner (1783-1852) a German pharmacologist, extracted an alcoholic β-glucoside, the active compound, from willow (*Salix*) bark, named salicin. The chemical analysis carried out in 1835 compared salicin to spiric acid, also effective against fever and pain, but extracted from a flowering plant, spirea. In 1853, the Strasbourg resident Charles Frédéric Gerhardt (1816-1856) synthesized an analogue of these molecules, the acetylsalicylic acid [101]. In 1897, Felix Hoffmann (1868-1946), a German chemist under the direction of Arthur Eichengrün (1867-1949) synthesized a pure form of acetylsalicylic acid [102]. This molecule was finally marketed by Friedrich Bayer (1825-1880) in 1899 under the name Aspirin®. Sir John Vane (1927-2004) deciphered the mechanisms of action of aspirin, and Charles Serhan (1954-) reported that aspirin favors the production of lipoxin A4, a lipid mediator involved in the resolution of inflammation. Lipoxin belongs to an important superfamily of lipidic pro-resolving mediators (resolvins, maresins, protectins) [103].

### Bloodletting

Hippocrates advocated bloodletting as another therapeutic approach to cure most diseases, including inflammatory disorders. Its use was supported by other erudite Greeks such as Erasistratus, Asclepiades of Bithynia, or Galen of Pergamon and later by the Roman scholar Aulus Cornelius Celsus, the Persian medical doctor Avicenna (10^th^ century), or the Spanish Jewish doctor Moïse Maïmonide (12^th^ century). All physicians of the kings of France were great supporters of bloodletting. Ambroise Paré (1509-1590), the physician of Charles IX, explained why he bled a young man 27 times in four days: “*I liked to mention this event, so that the young surgeon will not be too shy to draw blood when confronted to large inflammation*”. Laurent Joubert (1529-1583), the physician of Henri III, claimed that it was a way to get rid of the “bad blood” while the best was retained. Charles Bouvard (1572-1658) had probably prescribed 47 bloodlettings during the last 10 years of Louis XIII who died of Crohn’s disease at the age of 42 years. They used it even when their patients were still young. For instance, François Vaultier (1590-1652) bled the young Louis XIV at the age of 9 years when he had smallpox. Guy Patin (1601-1672), the dean of the School of Medicine in Paris for a brief period, declared: “*There is no remedy in the world that does so many miracles. I have bled my wife twelve times for a pleurisy, twenty times my son for a continuous fever and myself seven times for a cold*”. Even on the American continent, bloodletting was popular. Thus, on December 14, 1799, Georges Washington, probably suffering from pneumonia, died when 3.7L of blood were drained out of his body in one single day.

The first doctor to question the usefulness of bloodletting was Pierre Charles Alexandre Louis (1787-1872) who in 1835 considered this approach to have had very limited advantages. However, against his proposals were leading doctors such as François Broussais (1772-1838), who was alleged to have had spilled more blood than Napoleon on all battlefields! If the lancet was commonly used to remove blood from patients, then the use of leeches was another method. According to an estimate, 35 million leeches were used in France in 1830 alone. This trend went unabated until François-Vincent Raspail (1794-1878) questioned the method in 1845 saying: “*But why resort to violent and bloody means? Do you wish to calm fever? You will not succeed by bleeding* [(] *So leave your lancet there, it has made enough troubles since Hippocrates*” [104]. In 1856, in Great Britain, John Hughes Bennet (1812-1875) also concluded that there was no proven therapeutic advantage of bloodletting.

The other method used to prevent a severe inflammation and infection after a wound was cauterization supported by doctors such as Giovanni da Vigo (1450-1525) in Italy, and Paracelsus (1493-1541) in Switzerland. But Ambroise Paré, comparing the relative advantages of cauterization and the use of antiseptics, concluded that the latter were the best. In 1854, Florence Nightingale (1820-1910) advocated hygiene as a means to prevent infection during the Crimean war in order to limit the mortality of wounded soldiers. Of course, the main advocate of hygiene was Ignaz Semmelweis (1818-1865) who succeeded in 1847 to reduce mortality due to puerperal sepsis [105].

### Antiseptics

Since the Roman time and Galen, wine was used as an antiseptic. The fact that wound could be cleaned by wine was further advocated in Italy by Hugo Borgognoni de Lucas (1160-1257) and his son Teodorico Borgognoni de Lucas (1205-1298) and similarly in France by Henri de Mondeville (1260-1320). The word “antiseptic” was coined in 1750 by John Pringle (1707-1782), a Scottish physician, who studied numerous substances able to prevent putrefaction (e.g salt, lemon juice, pepper, mint, camphor and green tea). He even considered the systemic use of such antiseptics: "*whether putrefaction would be the only change made in the body by contagion, it would be easy to cure such fevers, at any period, by the use of acids or other antiseptics*" [106]. In France, Geneviève Thiroux d'Arconville (1720-1805) repeated many of Pringle’s experiments and confirmed his findings in 1761 [107]. Still in France, Louis Pomayrol (1819-1899) in 1853 and Casimir Davaine (1812-1882) in 1880 reported the antiseptic properties of extracts from leaves and bark of walnut when investigating the anthrax infection. Paul Erhlich (1854-1915) proposed different molecules derived from dyes to treat trypanosome infection with trypan red, and syphilis with salvarsan. Syphilis was also successfully treated by Metchnikoff with calomel, a mercury chloride. In parallel the discovery of antibiotics was made by numerous scientists who accidentally observed that fungal cultures (penicillium) had the capacity to prevent the growth of bacteria (Table 1). Few were able to understand the potential of such a discovery till Sir Alexander Fleming (1881-1955) published his observation on penicillin (see [108] for further description of antibiotics discoveries).

**Table 1.** Historical steps in antibiotics discoveries.

1869	Victor Feltz (1835-1893) & Léon Coze (1819-1896)	Penicillium prevents bacteria growth
1871	John Scott Burdon-Sanderson (1828-1905)	Penicillium prevents bacteria growth
	François Henri Hallopeau (1842-1919 )	Coined the word “antibiotic”
1874	Sir William Roberts (1830-1899)	Penicillium prevents bacteria growth
1874	Theodor Billroth (1829-1894)	Penicillium prevents bacteria growth
1876	John Tyndall (1820-1893)	Penicillium prevents bacteria growth
1893	Bartolomeo Gosio (1863-1944)	Mycophenolic acid as an antibacterial agent
1895	Vincenzo Tiberio (1869-1915)	Bactericial activity of fungal extracts
1897	Ernest Duchesne (1874-1912)	*In vivo* bactericidal activity of fungal extracts
1899	Rudolph Emmerich (1852-1914) & Oscar Löw (1844-1941)	First clinical use of antibiotics (pyocyanase)
1929	Sir Alexander Fleming (1881-1955)	Discovery of penicillin
1930	Selman Waksman (1888-1973)	Updated the word “antibiotic”
1935	Gerhard Domagk (1895-1964)	Discovery of sulfamidochrysoïdine, patented as Prontosil®
1935	Jacques Tréfouël (1897-1977) & Thérèse Tréfouël (1892-1978)	Discovery of the sulfamide
1936	Leonard Colebrook (1883-1967)	Clinical use of Protonsil® in puerperal fever
1939	René Dubos (1901-1982)	Discovery of tyrothricin
1940	Ernst Boris Chain (1906-1979)	Isolation and purification of penicillin
	Edward Abraham (1913-1999)	
	Howard Florey (1889-1968)	
	Norman Heathley (1911-2004)	
1942	Albert Schatz (1920-2005)	Discovery of streptomycin
1945	Benjamin Minge Duggar (1872-1956)	Discovery of aureomycin®

## Conclusion

As briefly summarized in this review, the inflammatory process was deciphered through the years by talented and inspired scientists. As soldiers and civilians bow down in front of the tomb of the Unknown Soldier, we should pay tribute to our glorious predecessors, even if sometimes their names have been forgotten [109].

## Footnotes

^1^ Nikolaï Gamaleïa finally made his career in Soviet Union and chaired an Institute that bore his name, now famous for the preparation of the Russian Sputnik V vaccine against SARS-Cov-2.
